# Metabolic factors in the regulation of hypothalamic innate immune responses in obesity

**DOI:** 10.1038/s12276-021-00666-z

**Published:** 2022-04-26

**Authors:** Andrew Folick, Rachel T. Cheang, Martin Valdearcos, Suneil K. Koliwad

**Affiliations:** Diabetes Center and Division of Endocrinology and Metabolism, Department of Medicine, University of California, San Francisco, CA USA

**Keywords:** Microglial cells, Obesity, Metabolic syndrome, Hypothalamus, Neuroimmunology

## Abstract

The hypothalamus is a central regulator of body weight and energy homeostasis. There is increasing evidence that innate immune activation in the mediobasal hypothalamus (MBH) is a key element in the pathogenesis of diet-induced obesity. Microglia, the resident immune cells in the brain parenchyma, have been shown to play roles in diverse aspects of brain function, including circuit refinement and synaptic pruning. As such, microglia have also been implicated in the development and progression of neurological diseases. Microglia express receptors for and are responsive to a wide variety of nutritional, hormonal, and immunological signals that modulate their distinct functions across different brain regions. We showed that microglia within the MBH sense and respond to a high-fat diet and regulate the function of hypothalamic neurons to promote food intake and obesity. Neurons, glia, and immune cells within the MBH are positioned to sense and respond to circulating signals that regulate their capacity to coordinate aspects of systemic energy metabolism. Here, we review the current knowledge of how these peripheral signals modulate the innate immune response in the MBH and enable microglia to regulate metabolic control.

## Obesity: The role of immune cells in the brain

Immune cells in the brain that contribute to metabolic dysfunction could offer a therapeutic target for treating obesity. In a review article, a team led by Martin Valdearcos and Suneil Koliwad from the University of California, San Francisco, USA, discuss the evidence linking microglia and other immune cells found in the brain’s hypothalamus region to the regulation of food intake and weight gain. The researchers describe how hormones, inflammatory molecules, nutrients, and microbiome-derived factors can all impact microglia in ways that affect how the immune cells interact with the neurons responsible for metabolic control. Although much remains to be learned about which aspects of microglial function are integral to obesity promotion, the authors argue that, in principle, drugs that target these immune cells could help combat excess weight gain and related metabolic disorders.

## Introduction

In many animal species, the central nervous system (CNS) plays a key role in sensing and controlling energy status. In this context, in mammals, the mediobasal hypothalamus (MBH) in particular has emerged as a master regulator of energy homeostasis. The MBH contains the median eminence (ME) and arcuate nucleus (ARC) and is particularly positioned to sense circulating factors that regulate metabolism. The ME and the ventromedial ARC are circumventricular organs (CVOs) that lack a true blood–brain barrier (BBB) formed by endothelial cells, resulting in exposure to circulating factors from the hypophyseal portal system^1,2^. The remaining portion of the ARC may be exposed to circulating factors via diffusion, and the ARC is also open to signals from the CSF via the infundibular recess^1^. Thus, the MBH is a critical brain region that integrates circulating signals from the periphery to regulate systemic metabolism.

Recent research has revealed that the regulation of hypothalamic function involves complex interactions between neurons and immune cells within the MBH^3^. Both the immune and nervous systems have evolved to sense intrinsic and extrinsic signals to coordinate a multicellular response that contributes to the maintenance of tissue homeostasis. Neuroimmune interactions are bidirectional, and immune cells produce signals such as cytokines, neuropeptides, neurotransmitters, and hormones to modulate neuronal functions^4,5^.

Microglia are specialized resident myeloid cells in the CNS, and there is growing evidence highlighting an expanding array of functions of these cells beyond their established roles in immunosurveillance and the clearance of cellular debris. For instance, microglia play a crucial role in shaping and maintaining neuronal circuits, which is a process called synaptic pruning, in which damaged or unnecessary synapses are eliminated to maintain synaptic homeostasis^6,7^. Furthermore, microglia also produce various neurotrophic factors to properly regulate neuronal excitability and promote the differentiation and survival of neurons^8,9^. Therefore, microglia are central players that contribute to neuroimmune interactions to maintain brain homeostasis.

With respect to the hypothalamus, we showed that microglia in the MBH can sense increased consumption of saturated fats and transduce this signal to instruct local neurons engaged in controlling hunger and satiety^10^. Moreover, microglial activation alone is sufficient to stimulate food intake and body weight gain in adult mice^11^. Hypothalamic inflammatory pathways are rapidly activated in response to the initiation of high-fat diet (HFD) feeding, far before any significant weight gain manifests, suggesting that this response is causative in the pathogenesis of obesity. As such, a better understanding of the dietary, metabolic, and immunological factors that drive microglial activation in the MBH will be critical in potentially developing new therapeutic strategies for obesity and related metabolic disorders.

**Fig. 1:**
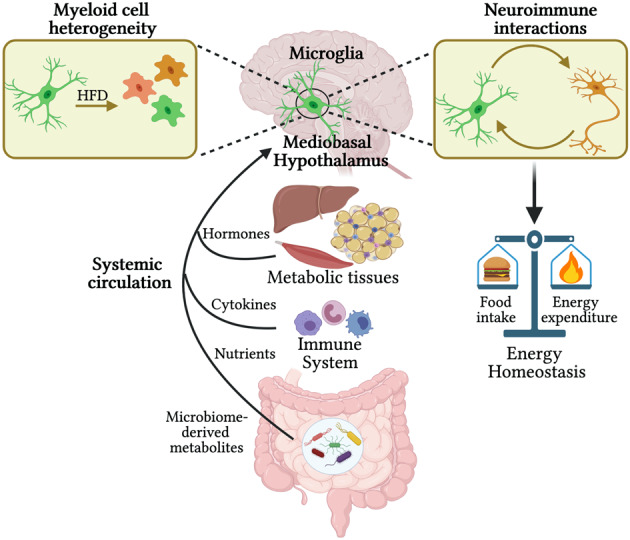
Systemic factors impacting myeloid cells in the MBH to regulate hypothalamic control of energy homeostasis. Myeloid cells in the MBH are activated by HFD, which affects hypothalamic neuronal activity to regulate energy homeostasis. Myeloid cells in the MBH are exposed to the systemic circulation and are thus positioned to sense and respond to circulating factors. Diet-induced obesity (DIO) is characterized by changes in nutritional signals, inflammatory cytokines, metabolic hormones, and microbiome-derived molecules, which may modulate MBH microglial function. Figure created with BioRender.com.

The signaling mechanisms connecting consumption of a HFD to the innate immune response in the MBH remain largely unknown, but ongoing work to elucidate connections between systemic signals and the MBH provides clues as to how such signals may be relayed (Fig. 1). One possible mechanism is circulating nutritional or hormonal signaling molecules that are either directly obtained from the diet or generated in the periphery in response to a HFD and received by the MBH. For example, the enteric immune system can also release cytokines directly into the circulation, and these cytokines can reach the brain and modulate the neuronal activity of CVOs, including the MBH^12^. In addition, circulating immune cells have been shown to respond to a HFD and can themselves be recruited to sites of damage or inflammation^13^.

Moreover, peripheral neurons can receive and convey long-distance information through complex neural circuits, forming a brain–gut axis in which visceral and vagal afferent fibers relay local signals from the gut to the brain^14,15^. In the brain, microglia express receptors for and have been shown to respond to neuropeptides and chemokines in addition to classical neurotransmitters^16^. However, vagal afferent fibers are not known to directly innervate the MBH and thus would require a more complex neuronal circuit to directly impact microglia in the MBH. Indeed, vagal afferent fibers synapse onto neurons in the nucleus tractus solitarius (NTS), which in turn has reciprocal connections with metabolic neurons in the hypothalamus^14,17^. Manipulation of the vagus nerve in mice and humans has well-known effects on metabolism, but little is known about the impact of vagal inputs on the state of hypothalamic immune cells^18^. Given the paucity of literature in this area, we will instead focus this review on circulating factors that could allow nutritional stimuli to induce hypothalamic innate immune responses.

While the evidence supporting the causal role of the hypothalamic innate immune response in obesity comes primarily from rodent models, several studies have suggested that hypothalamic gliosis is relevant to human obesity. For example, the activation of immune cells within a specific brain area results in increased regional water content, which can be detected by quantitative MRI techniques. Given this, multiple MRI techniques to assess brain water content, including T2 relaxation time^19,20^, diffusion tensor imaging^21^, and proton density imaging^22^, have revealed a potential association between hypothalamic inflammation and BMI, central adiposity, and metabolic syndrome in humans^20,22^.

Obesity is associated with hypothalamic hypogonadism in men, and MRI indicators of hypothalamic gliosis are also inversely correlated with plasma testosterone levels^23^. Moreover, these imaging markers highlight a potential reduction in hypothalamic inflammation in obese individuals who experience weight loss following Roux-en-Y gastric bypass surgery^24,25^. Overall, these studies provide evidence supporting the clinical relevance of hypothalamic inflammation^24,25^. It is, however, important to note that MRI-based indicators of inflammation in the MBH could reflect increased activation of microglia and/or astrocytes. However, human hypothalamic samples examined postmortem showed that elevated BMI is indeed associated with specific increases in microglial soma size, along with reductions in the extent of microglial ramifications, which are both morphological hallmarks of inflammatory activation^26,27^ that underscore the importance of microglia in human energy balance.

## Myeloid cell heterogeneity in the mediobasal hypothalamus

The myeloid compartment of the CNS includes a heterogeneous population of tissue-resident macrophages, including microglia in the brain parenchyma and macrophages and dendritic cells in perivascular, meningeal, and choroid plexus compartments^28,29^. Microglia are the most abundant myeloid cell type in the CNS and the predominant cell type within the brain parenchyma^28^. CNS myeloid cells express numerous overlapping markers, and thus a major obstacle in the field has been the inability to discriminate between resident “homeostatic” microglia and the variety of other myeloid cell types in the brain. New technical approaches for single-cell profiling, however, have recently overcome this obstacle and revealed remarkable functional complexity within the CNS myeloid compartment in the context of normal health and disease^30^. Resident microglia are yolk sac-derived cells and self-renew within the brain throughout life, and there is increasing evidence that these cells function differently in the brain than bone marrow (BM)-derived macrophages, which infiltrate the brain under conditions of CNS injury and disease^31,32^. Microglia are long-lived cells and depend highly on signals from their tissue environment to maintain identity, but there is growing evidence that even true microglia are not monolithic, exhibiting spatial heterogeneity and varying functional states dictated by anatomical location and physiological or pathological factors both in the rodent and human brain^33^. These findings have important therapeutic implications because they suggest that targeting a specific microglial subset or state may require strategies that are specific to a given brain region. Moreover, drug targeting of most parenchymal microglia is limited by the BBB. With this in mind, the MBH may provide an interesting therapeutic opportunity, as the ME and ventromedial ARC contain highly fenestrated blood vessels, which facilitate relatively open communication with the periphery^1,2^. Thus, myeloid cells in the MBH are potential targets that may be accessed by modulators delivered enterally, parenterally, or intranasally^34^. Furthermore, bloodborne (e.g., monocyte-derived) myeloid cells are recruited to the brain during pathological states that are associated with compromised BBB integrity, and these cells, once recruited into the brain parenchyma, have the capacity to gain key microglia-like characteristics^28,35^. As such, these infiltrating cells are also recognized as targets for therapeutic intervention in neurological disease^36,37^.

We used detailed immunofluorescence histochemistry and BM lineage tracing to show that the HFD-induced hypothalamic innate immune response actually involves diverse myeloid cell phenotypes and functional states with unique spatial distributions within the MBH^11^. Moreover, a recent study showed that inducible nitric oxide synthase (iNOS) activation in hypothalamic LysM^+^ macrophages contributes to the diet-driven immune response within the MBH by increasing local vascular permeability and lipid influx^38^. These LysM^+^ macrophages apparently accumulate in the MBH in response to a HFD through local proliferation, without the contribution of BM-derived cells^39^. Understanding the functional heterogeneity of individual myeloid cell types in the MBH, both resident and infiltrative, and the specific signaling pathways that are regulated by diet in these populations will be essential in developing selective cell-based and/or immunotherapeutics to correct diet-induced hypothalamic dysfunction.

## Circulating factors that regulate hypothalamic innate immune responses

Translating knowledge of myeloid cells in the MBH for therapeutic purposes will require understanding which potential circulating factors influence the polarization of these cells. It has long been known that hypothalamic nutrient sensing is critical for the regulation of energy balance, with recent work focusing on the hypothalamic sensing of nutritional signals, especially glucose and lipids^40^. Hypothalamic neurons are well known to be regulated by circulating metabolic signals, and both hypothalamic neurons and glial cells have been shown to respond to lipid signals that regulate both food intake and energy homeostasis^41^. However, the comparative roles of the neuronal and nonneuronal compartments in this regard have not been fully elucidated. In addition to specific nutrients, this section will review the circulating hormonal, inflammatory, and microbiome-derived factors that may regulate hypothalamic immune activation in the development of diet-induced obesity (DIO).

### Hormonal regulation of the hypothalamic innate immune response

There is an expanding list of peripherally generated hormones that regulate food intake and metabolism and act on hypothalamic signaling pathways^15^. However, the effects of these hormones, which are derived from the gut, adipose tissues, and muscle, on hypothalamic immune activation remain poorly characterized. Here, we review what is known about hormonal regulation of the hypothalamic innate immune response.

#### Leptin

Leptin is a peptide hormone secreted primarily by white adipocytes that is central to the regulation of body weight and energy expenditure^42^. While previously thought to be expressed predominantly by specific neuronal populations, including POMC and NPY/AgRP/GABA (NAG) neurons, leptin receptor mRNA has also been found in astrocytes and microglia^43^. Initial studies showed that while both leptin-deficient (*ob/ob*) and leptin receptor-deficient (*db/db*) mice are obese, this phenotype alone is not sufficient for mice in either model to develop microgliosis in the MBH. However, *ob/ob* mice develop hypothalamic microgliosis in a manner similar to that of wild-type controls when fed a HFD^44^. Taken together, these early results suggested that dietary excess, rather than obesity per se, mediates the activation of MBH microglia and that leptin-dependent signaling in the brain is not a dominant determinant of this response. However, there is more recent evidence suggesting that microglial reactivity may also be modulated by leptin signaling. For example, providing *ob/ob* mice with leptin replacement is sufficient to increase microglial numbers in the MBH^44^. Moreover, pretreating cultured microglia with leptin potentiates their inflammatory response to lipopolysaccharide (LPS), and leptin increases the expression of tumor necrosis factor alpha (TNFα) and interleukin-1 beta (IL‐1β) in primary hypothalamic microglia^44,45^. Finally, myeloid-specific deletion of the leptin receptor led to increased weight gain in mice fed a standard chow diet, although this was predominantly due to increased lean mass^46^. Notably, this study was performed in a noninducible genetic model, pointing to the possibility that the resulting hypothalamic and metabolic phenotypes might be influenced by impairments in synaptic pruning by microglia during embryonic and early-life development^46^.

#### Adiponectin

Adiponectin (APN) is an adipokine that is almost exclusively secreted by adipocytes^47^, and circulating levels of its high molecular-weight multimeric isoform are decreased in individuals with visceral adiposity and insulin resistance^48^. APN-deficient mice are resistant to HFD-induced obesity^49^. Exploration of this phenotype has revealed that APN is able to cross the BBB and stimulates food intake via activation of the two APN receptors (ADIPOR1 and ADIPOR2) in the ARC; in rats, these receptors have been shown to be expressed not only in POMC and NAG neurons but also in microglia and astrocytes^50,51^. Notably, APN-deficient mice displayed enhanced microglial proliferation and inflammatory cytokine expression in both the hypothalamus and hippocampus following LPS injection, and this hyperresponsiveness was suppressed by i.c.v. injection of globular APN^52^. Treatment of mice fed a HFD for 4 weeks with systemic (i.p.) APN was associated with decreased microglial activation, as indicated by morphological changes, and decreased expression of inflammatory cytokines in the hypothalamus; however, this effect was not associated with an alteration in body weight^51^. Whether APN acts directly on hypothalamic microglia in vivo has not been studied.

#### Glucagon-like peptide 1

Glucagon-like peptide 1 (GLP-1) is a peptide hormone that is primarily secreted by enteroendocrine L-cells in the intestine^53^. Several GLP-1 receptor agonists have now been approved for the treatment of diabetes and/or obesity, and the anorectic effects of GLP-1, although pleiotropic, are increasingly linked to agonism of the GLP-1 receptor (GLP-1R) in multiple brain regions^54^. Indeed, GLP-1R is expressed by neurons, astrocytes, and microglia^55,56^. Within the hypothalamus, GLP-1 stimulates the activity of specific GLP-1R-expressing neuronal populations, including POMC neurons. However, GLP-1R expression by hypothalamic neurons was paradoxically dispensable in GLP-1 treatment to induce weight loss or improve glucose tolerance in mice^57,58^. Intriguingly, GLP-1 has direct anti-inflammatory effects on microglia, decreasing their secretion of proinflammatory cytokines^59^. Systemic administration of the GLP-1 analog exendin-4 and, in a separate study, liraglutide decreased microglial activation within the MBH of mice fed a HFD^44,60^. However, whether the beneficial effects of GLP-1R agonists are at least partly mediated by the modulation of hypothalamic microglial activation remains to be studied.

Although systemic GLP-1 can cross the BBB^61^, it is notable that GLP-1-producing (PPG) neurons in the NTS were recently shown to be another major source of GLP-1 within the brain^62^. Ablating NTS PPG neurons did not affect body weight or food intake on a standard chow diet but markedly increased feeding behavior following a fast, when food consumption rates are typically high^62^. Nevertheless, whether GLP-1 produced in the periphery or in the brain is more dominant in modulating the hypothalamic response to HFD has not been assessed.

#### Ghrelin

Ghrelin is an orexigenic hormone produced predominantly by the gut, but it is also expressed in other tissues, including the ARC^63,64^. While ghrelin has potent orexigenic effects that are mediated by NAG neurons in the ARC, it also has anti-inflammatory effects on microglia in vitro and in models of experimental autoimmune encephalitis^65^. In one study, pretreatment of mice with a single injection of purified ghrelin in its active acylated (n-octanoyl) form reduced the hypothalamic inflammatory response to a single day of HFD feeding^66^. Taken together, these results suggest that ghrelin signaling may play a role in disassociating hypothalamic microglial activation from the effects of dietary excess or perhaps even restraining it.

#### Estrogens

Estrogens may also serve to modulate both hypothalamic neurophysiology and the interaction between MBH neurons and local microglia. The hypothalamic microglial response to the consumption of a HFD exhibits striking sexual dimorphism in rodents, with males having higher levels of inflammatory cytokines and greater microglial activation than females^67,68^. There is evidence suggesting that both cell-intrinsic and environmental factors impact microglial function in a sexually dimorphic manner, and these factors may help determine sex-specific differences in susceptibility to neurological disease^69^. Ovariectomy-induced estrogen deficiency increases microglial activation in female mice^68^. In contrast, both 17-α-estradiol (17αE2) and 17-β-estradiol (17βE2) have anti-inflammatory effects on microglia in vitro, and 17αE2 treatment reduced aging-associated hypothalamic microglial activation in male mice and reduced microglial activation and accumulation within the MBH of ovariectomized female mice fed a HFD^70,71^. However, data also suggest that microglia show innate sexual dimorphism, independent of hormonal inputs^72^. Future studies should examine how such innate elements interact with environmental (hormonal, dietary) factors to functionally modulate MBH microglia.

#### BAIBA

β-aminoisobutyric acid (BAIBA) is a myokine that is increased by physical activity and was shown to mediate the benefits of exercise on obesity-related metabolic risk factors in mice but with only modest effects on weight gain itself^73,74^. A regimen of moderate exercise decreased the MBH microgliosis induced by the consumption of a high-carbohydrate HFD in LDL receptor-deficient mice^75^. Similarly, BAIBA treatment for 8 weeks lowered the hypothalamic mRNA levels of inflammatory genes and reduced the number of hypothalamic microglia in mice that had already been fed a HFD for 16 weeks, although it did not reverse weight gain^73^.

In summary, there are currently no data directly implicating circulating hormonal signals in driving the mechanism by which dietary excess causes hypothalamic innate immune activation. However, several hormones may play roles in modulating this response, with leptin being potentially proinflammatory, and most others have roles in restraining hypothalamic immune activation.

### The hypothalamic innate immune response to systemic inflammation

Obesity can induce chronic systemic inflammation and increase the circulating levels of inflammatory molecules, including LPS, interleukin-6 (IL-6), C-reactive protein (CRP), plasminogen activator inhibitor 1 (PAI-1), and serum amyloid A (SAA), in certain individuals^76^. The levels of these factors can then rise in the MBH as well. In addition, the levels of other cytokines, such as TNFα and IL-1β, are known to be increased in the MBH in obesity; however, these cytokines are most likely increased due to local production rather than import from the systemic circulation. Microglia express receptors and machinery that are involved in many aspects of innate immune signaling^77^, raising the possibility that circulating and/or local inflammatory mediators may play a role in regulating the polarization of MBH microglia in obesity. However, microglial activation in the MBH occurs rapidly in response to a HFD, and a recent study demonstrated a response as early as 6 h after exposure to a HFD^78^. In contrast, although some studies have demonstrated an increase in circulating inflammatory cytokines after a single high-fat meal, a recent systematic review showed that only IL-6 was consistently increased after one high-fat meal in healthy individuals^79^. Overall, these studies raise some doubt as to whether circulating levels of inflammatory factors rise fast enough in response to the initiation of a HFD to represent viable sources of factors that then stimulate microglial activation within the MBH. The alternative is to consider that noninflammatory factors (nutrients, microbiome-derived factors, hormones, etc.) alter the functional states of MBH microglia and that these effects precede any systemic inflammatory changes that may occur later on. Regardless, it seems likely that circulating cytokines and other immune-related signals may help regulate and modulate chronic diet-associated hypothalamic innate immune activation. Some potential signals are reviewed here.

#### LPS

LPS levels in the circulation can be increased by HFD consumption and remain elevated in obesity, possibly due to increased gut permeability^80,81^. Indeed, systemic administration of a low dose of LPS to mimic the levels seen upon chronic consumption of a HFD was sufficient to induce weight gain and insulin resistance in mice^81^. How peripheral LPS impacts brain function is unclear, as most of the brain is relatively protected against peripheral LPS, with an estimation that only 0.025% of an i.v. injection reaches the brain parenchyma^82^. A recent study showed that peripherally injected LPS is present, however, within CVOs, including the ME and that the transport of LPS into CVOs may be mediated by lipoproteins^83^. Thus, CVOs may function as sensors of peripheral LPS. Moreover, a single i.p. injection of LPS in mice induced the proliferation of microglia in hypothalamic CVOs, including the ME and ARC^84^. However, a recent study showed that while peripheral LPS injection caused microglial activation in lean animals, intermittent LPS injection decreased HFD-induced microglial activation and increased MBH astrogliosis^85^.

Toll-like receptor 4 (TLR4) is the cell-surface receptor for LPS and is expressed most highly by myeloid cells in the CNS, including in the hypothalamus^86,87^. Whole-body TLR4 knockout has shown variable effects on the response to a HFD in different studies, providing protection from weight gain in some but not in others^88–90^. One explanation for this discrepancy is that TLR4 deletion in different tissues may have variable effects on DIO. Intriguingly, the transplantation of TLR4-deficient BM also had variable effects on HFD-induced weight gain^91–94^. More recently, hepatocyte-specific targeting of TLR4 ameliorated hepatic steatosis and improved insulin resistance but did not affect weight, while macrophage-specific targeting of TLR4 did not protect mice from DIO, insulin resistance or hepatic steatosis^95^. In the hypothalamus, TLR4 targeting through stereotactic injection of TLR4 shRNA lentiviral particles into the ARC decreased microglial accumulation and body weight gain during 4 weeks of HFD feeding^96^. Daily intraperitoneal injection of a TLR4-inhibiting antibody to animals on a HFD decreased weight gain and the expression of inflammatory cytokines in the MBH^97^. However, microglia-specific TLR4 deficiency has not been examined in a mouse model of DIO. It has been previously hypothesized that TLR4 may also mediate innate immune activation in DIO by functioning as a direct receptor of saturated fatty acids (SFAs). However, a recent study demonstrated that while TLR4 may be required for SFA-induced innate immune activation, it is not an SFA receptor. Rather, TLR4 is required for priming cells for SFA-induced inflammatory responses^98^. The question remains whether increased LPS levels in the context of DIO are necessary for TLR4-dependent priming of microglia to drive hypothalamic inflammatory responses in vivo.

#### IL-6

IL-6 is a cytokine associated with obesity and insulin resistance. Indeed, acute IL-6 infusion is sufficient to induce insulin resistance in mice^99^. Interestingly, myeloid-specific depletion of the IL-6 receptor (IL-6R) causes a shift toward M1 macrophage polarization in liver, white adipose tissue, and brown adipose tissue. Moreover, this polarization shift in macrophage populations is associated with impaired glucose homeostasis^100^. However, the effects of chronically increased systemic levels of IL-6 are pleiotropic and include some beneficial consequences, depending on the model^101^. For example, IL-6-deficient mice paradoxically develop maturity-onset obesity, and central IL-6 administration was able to increase energy expenditure in this model^102^.

When delivered into the CNS (i.c.v. injection), IL-6 decreased feeding and improved glucose homeostasis, but deleting IL-6R in neurons did not block these effects^103^. Moreover, CNS delivery of an antibody that inhibits IL-6 signaling blocked the anti-inflammatory effect of exercise on the hypothalamus^104^. Glycoprotein 130 (gp130) is the transmembrane receptor for IL-6 trans-signaling, which is engaged by IL-6 binding to soluble IL-6R. Targeting gp130 in neurons of the paraventricular nucleus (PVN) of the hypothalamus blocked the metabolic effects of central IL-6 administration^103^. The cellular source of soluble IL-6R that mediates IL-6 trans-signaling in the brain has not been found, but IL-6R mRNA is most highly expressed by microglia^86^. Interestingly, IL-6 trans-signaling was also essential in the neuroprotective effects of microglia in a model of traumatic brain injury^105^. IL-6 is released at high levels by exercised muscle^106^; however, multiple brain cell types may synthesize IL-6 locally^107^. Whether systemic and/or local IL-6 are involved in the regulation of hypothalamic function remains unknown. Further functional resolution of cell- and tissue-specific IL-6 signaling pathways will be important in determining why acute and chronic manipulations of IL-6, whether systemically or specifically in the CNS, produce wide-ranging and seemingly paradoxical effects on obesity.

#### Plasminogen activator inhibitor 1

Plasminogen activator inhibitor 1 (PAI-1) is produced by adipose tissues, and the levels are elevated in both adipose tissues and plasma in obesity^108^. Both genetic deletion and pharmacologic inhibition of PAI-1 protect against HFD-induced increases in food intake and body weight^109,110^, and this effect was found to be mediated at least in part by impaired leptin signaling in the hypothalamus^109^. The effects of PAI-1 on MBH immune activation have not been explored; however, PAI-1 has been shown to promote microglial migration and regulate phagocytosis^111^.

In summary, there are intriguing data suggesting that circulating immune-related factors may modulate the hypothalamic innate immune response by acting on microglia. However, detailed and systematic studies to determine how these factors might coordinately mediate hypothalamic dysfunction and the development of DIO have not yet been performed.

### Dietary composition and regulation of the hypothalamic innate immune response

The effects of specific macronutrient proportions on obesity remain a subject of controversy and ongoing investigation. Most diets used to produce obesity in rodents are high in saturated fat; however, these diets contain significant calories from sugar as well. A recent systematic evaluation of the effect of macronutrient composition on body weight regulation showed that in mice, increased dietary fat content but not protein or carbohydrate content was associated with increased food intake and weight gain, plateauing with diets in which 60% of calories come from fat^112^. Importantly, this systematic comparison was performed in male C57BL/6J mice, which are sensitive to both DIO and HFD-induced hypothalamic innate immune activation, and it is unclear how transferrable these findings are to other mouse strains. Moreover, interpersonal differences in obesity-susceptibility genotypes have been shown to interact with dietary macronutrient composition to produce an integrated obesity risk profile in humans^113,114^. Thus, rodent experiments exploring the effects of macronutrient composition on aspects of DIO must be interpreted with these key limitations in mind.

Large-scale systematic evaluations of the effects of macronutrient composition on the activation state of hypothalamic immune cell types have not been performed, and most studies used standard HFDs varying from 40 to 60% of caloric intake from fat with the source of fat predominantly being either milk fat or animal lard, which are both high in saturated fat (Table 1). Common diets that have been demonstrated to induce hypothalamic innate immune activation include D12451 (Research Diets, 45% kCal fat, lard), 88137 (Teklad, 42% kCal fat, milk fat), and D12492 (Research Diets, 60% kCal fat, lard) (Table 1)^115–117^. These diets have been shown to increase the levels of hypothalamic inflammatory cytokines and promote microglial activation and also lead to increased caloric intake. This raises the question of whether increased caloric intake per se causes hypothalamic immune activation. However, calorically matched pair-feeding experiments with distinct HFDs demonstrate that consuming excess dietary fat is sufficient to cause weight gain and hypothalamic innate immune activation independent of overall caloric consumption^117^.

**Table 1:** Dietary composition of high-fat diets used for studying hypothalamic innate immune response.

Diets ^(Manufacturer)^	caloric content (kcal/g)	%Fat (kcal)	%Carb (kcal)	%Prot (kcal)	Fat source	% SFA	microglial changes		MBH inflammatory cytokines			Reference
							M	N	TNF	IL1β	IL6	
D12492^(RD)^	5.21	60	20	20	Lard	33%	+	+	+	+	+	^27,38,66,68,71^
D12451^(RD)^	4.7	45	35	20	Lard	30%	+	+	+	+	+	^11,19^
D12331^(RD)^	5.56	58	25	17	Coconut oil	82%	+	+	+	+	+	^140^
TD.88137^(Envigo)^	4.5	42	43	15	Milk fat	62%	+	+	+	+	+	^10^
D11012901^(RD)^	4.4	32	51	17	Coconut oil	75%	NR	NR	+^a^	−^a^	−^a^	^118^
D11061301^(RD)^	4.4	32	51	17	Butter	60%	NR	NR	−^a^	+^a^	+^a^	^118^
D11012902^(RD)^	4.4	32	51	17	Olive oil	20%	NR	NR	−^a^	−^a^	−^a^	^118^
HCHF1^(NR)^	5.56	58	25	17	Coconut oil	NR	+	+	NR	NR	NR	^120^
HCHF2^(NR)^	5.08	62	19	19	Beef tallow	NR	+	+	NR	NR	NR	^120^
LCHF1^(Kliba Nafag)^	6.17	79	2	19	Beef tallow	68%	−	+	NR	NR	NR	^120^
LCHF2^(Kliba Nafag)^	7.2	93	2	6	Beef tallow	60%	−	−	NR	NR	NR	^120^

*RD* Research Diets, Inc., *OY* Oriental Yeast Co., *M* morphology, *N* number, *NR* not reported.

^a^Inflammatory cytokines compared amongst HFD compositions without low-fat control.

Several studies have also examined the effects of the amount and source of saturated fat in the diet on hypothalamic innate immune activation. One study compared the effects of diets that were low in saturated fat (corn and olive oil, 20% of total fat calories as saturated fat), high in saturated fat of plant origin (coconut oil, 75% saturated fat), or high in saturated fat of animal origin (butter, 60% saturated fat) (Table 1)^118^. While there was no difference in adipose tissue inflammatory markers, animals on both types of high-saturated fat diets showed increased levels of inflammatory cytokine expression in the hypothalamus^118^. In another comparison of diets with similar macronutrient compositions, a diet with elevated levels of saturated fat caused a more rapid increase in food intake than did one with lower saturated fat content^119^. Moreover, in a direct comparison of two diets, one of animal origin and one containing olive oil, which is high in monounsaturated oleic acid, only the diet with animal fat caused increased inflammatory cytokine expression and myeloid cell immunostaining in the MBH, although the total fat content of both diets was matched at 36% of overall calories^97^. Thus, the available evidence suggests that high-saturated fat intake is required for a HFD to induce hypothalamic innate immune activation in mice. Furthermore, it was shown that even very short-term (1–6 h) exposure to excess dietary saturated fat is sufficient to induce changes in MBH microglia^78^. These studies suggest that the composition of consumed fat, rather total fat intake, regulates immune activation in the hypothalamus in promoting DIO. The rapidity of this response is consistent with a direct effect of dietary saturated fat.

A recent study explored the role of dietary carbohydrates in the pathogenesis of MBH innate immune activation using high-fat diets with either low or high amounts of carbohydrates. Neither of the two very low carbohydrate diets used in this study, a nonketogenic diet (LCHF1) and a ketogenic diet (LCHF2), were able to induce MBH microgliosis or weight gain (Table 1)^120^. Another low carbohydrate, high-fat diet (D11111601, Research Diets) suppressed both the increased microglial cell division seen in the MBH and the increased food intake produced by the consumption of a standard HFD (D12492)^121^. These studies suggest that very low carbohydrate intake can protect against HFD-induced MBH innate immune activation.

Our group has explored the specificity of saturated fat in mediating the MBH innate immune response by direct gavage into the stomachs of mice. Gavage with milk fat, which is high in palmitic acid, but not olive oil reproduced the MBH microglial response observed following the consumption of a HFD enriched in saturated fat^10^. Moreover, the inflammatory effects of dietary fats administered into the brain (i.c.v.) are linked to specific SFAs^97,122^. In contrast, i.c.v. administration of oleic acid actually decreased food intake and hepatic glucose production compared with the control^123^. Hypothalamic sensing of oleic acid appears to be mediated by the regulation and sensing of intracellular long-chain fatty acyl-CoA levels by MBH neurons^124^. There is no known role for MBH microglia in the hypothalamic sensing of oleic acid; however, short-term (3 days) HFD feeding abolishes the metabolic and anorexigenic effects of i.c.v. oleic acid administration^125^. Of course, i.c.v. injection is an artificial model, but there is evidence that the hypothalamic lipid composition reflects dietary fat composition over time. Radiolabeled palmitic acid delivered enterally and lipoproteins injected i.v. were both able to rapidly enter the MBH^10,126^.

Further supporting a direct role for SFAs in functionally regulating hypothalamic microglia, multiple groups have demonstrated that saturated but not unsaturated FAs have proinflammatory effects on cultured microglia^127^. We showed that treating organotypic hypothalamic slices with palmitic acid but not oleic acid initiates an inflammatory response in the hypothalamic slice, which requires the presence of microglia^10^. Indeed, oleic acid cotreatment abrogated the proinflammatory response of these slices to palmitic acid treatment. Thus, saturated fat itself may function as an immunomodulatory signal that is delivered via the systemic circulation to mediate hypothalamic innate immune activation in DIO.

### Microglia are regulated by microbiome-derived factors

The gut microbiome is increasingly accepted to be an environmental factor linked to obesity and associated metabolic disorders. The diverse gastrointestinal microbial community participates in energy extraction from food, host lipid metabolism, immune responses, and endocrine function^128^. These observations were made using germ-free (GF) mice that were maintained in a sterile environment without a gut microbiome. GF mice are leaner than specific pathogen-free (SPF) mice despite consuming more calories. Furthermore, transplanting the microbiota from SPF mice into GF mice increased body fat by 60% within 2 weeks^129^. Indeed, the gut microbiome regulates both host energy expenditure and feeding behavior^130^. In humans, obesity, by contrast, produces a gut microbiome that is less diverse and consumes less energy than that of lean individuals^131^. Genetically obese mice (*ob/ob*) have a higher proportion of intestinal *Firmicutes* and 50% fewer *Bacteroidetes* and a parallel enrichment of microbial genes involved in polysaccharide degradation than in the microbiomes of lean siblings^132^.

The gut microbiome also controls the activity of CNS myeloid cells. Disruption of the gut microbiome resulted in significant alterations in microglial function in both GF mice and mice in which the microbiome was ablated by antibiotic treatment^133,134^, indicating that microglia may continuously sense inputs emanating from the gut microbiome. For instance, short-chain fatty acids (SCFAs) are the main metabolites produced by the bacterial fermentation of dietary fiber in the gastrointestinal tract, and these SCFAs are rapidly absorbed by colonic cells. SCFAs influence gut–brain communication and brain function directly or indirectly through immune, endocrine, and vagal pathways^135^. Although SCFAs have been shown to protect against DIO in mice^136^ and overweight humans^137^, the underlying mechanisms are unclear. SCFAs are important regulators of innate immune responses and were recently shown to be involved in regulating microglial function. Indeed, GF mice display globally defective microglial phenotypes, leading to impaired CNS immune responsiveness. Remarkably, aspects of normal microglial homeostasis can be restored when GF mice are supplemented with SCFAs in their drinking water^133^. Furthermore, recent work suggests that microglial activity is directly regulated by the metabolites of dietary tryptophan metabolism produced by commensal gut flora and that this response controls a downstream inflammatory response in astrocytes^138^.

The gut microbiota is also affected by circadian rhythms, with some microbes showing diurnal fluctuations in their relative abundance and activity^139^. Microglia in the MBH are also affected by the intrinsic circadian clock, with microglial numbers and activity being increased in the dark relative to the light phase in mice under standard housing conditions^140^. However, it remains unknown whether any aspect of circadian regulation of microglial numbers or activity, particularly within the hypothalamus, is a function of the circadian aspects of the gut microbiome. Building on a wealth of information highlighting microglia and other CNS myeloid cell types as crucial mediators linking the gut microbiome to pathological outcomes in the context of several neurodegenerative disease models^141^, future studies will need to comprehensively evaluate how the composition, secretome, and circadian regulation of the gut microbiome interact to regulate hypothalamic control of energy and glucose homeostasis.

## Novel tools to target distinct CNS immune cell subsets

The most commonly used mouse line to manipulate microglia is based on the Cx3cr1 (encoding fractalkine receptor) promoter, and Cx3cr1^CreER^ mouse strains have substantially advanced the microglial field. However, *Cx3cr1* gene expression within the CNS is not restricted to microglia, as it is also expressed in nonparenchymal macrophages at CNS border locations^142^. Furthermore, Cx3cr1 has been shown to be expressed by long-lived peripheral tissue-resident myeloid cell types^143,144^. Therefore, the results obtained using this mouse model in reference to microglia should be interpreted with caution. Moreover, this mouse line was generated based on knock-in strategies in which the endogenous gene is replaced, and there is evidence that *Cx3cr1* haploinsufficiency affects microglial function^145,146^. Thus, more specific tools to label and manipulate microglia are needed to further understand how this cell type contributes to brain health and disease.

Recent advances in RNA sequencing and other profiling technologies have revealed new insights into CNS myeloid cells, including a greater degree of heterogeneity than had been previously recognized. This information has been used to attempt to develop better mouse tools. Single-cell RNA-sequencing studies identified distinct core signature genes for microglia, such as *Sall1*, *Tmem119, P2ry12*, and *Hexb*. Sall1, a conserved transcriptional regulator, emerged as a marker to distinguish *bona fide* parenchymal microglia from other brain myeloid cells^147^. However, Sall1^CreER^ transgenic mice show prominent recombination in the neuroectodermal lineage^148^. Transmembrane protein 119 (Tmem119), a cell-surface protein, was also recently identified as a novel microglia-specific marker that is not expressed by peripheral myeloid cells^149^. However, a recent targeting approach revealed ectopic expression of Tmem119 in CD34+ vessels in the CNS and leptomeningeal pial cells^150^. P2ry12^CreER^ transgenic mice have been recently described^151^. Since *P2ry12* expression has been shown to be downregulated upon microglial activation^152^, this new mouse line will be particularly useful for tracking microglia during disease and development. Furthermore, hexosaminidase subunit beta (*Hexb*) is a highly enriched microglial gene with only minimal expression by other brain myeloid cell types, even in models of neuroinflammatory diseases. The *Hexb* locus has been used for the generation of two promising transgenic mouse lines: the Hexb^tdT^ and Hexb^CreERT2^ strains^153^.

Despite these impressive recent advances, the use of single promoter activity could be insufficient to specifically target a desired population of microglia. To overcome this problem, a second dimension of recombination, such as using the “split-Cre” system where Cre recombinase expression is regulated by coincidental activity of two different promoters, increasing the specificity of Cre-mediated DNA recombination^154^, could be highly effective. Such a binary transgenic system involving complementation-competent NCre and CCre fragments whose expression is driven by distinct promoters was recently used to specifically target parenchymal microglia using Sall1^NCre^:Cx3cr1^CCre^ mice and perivascular macrophages using Lyve1^NCre^:Cx3cr1^CCre^ mice^155^ simultaneously.

Such new and more specific tools will be necessary for further experiments to identify the specific myeloid cell populations in the MBH that are responsible for the initial and ongoing hypothalamic immune activation in response to dietary excess.

## Concluding remarks and future directions

The hypothalamic innate immune response is a component of DIO, and evidence suggests that myeloid cells, particularly microglia, are critical cellular drivers of this process. Recent studies have focused on the role of myeloid cells within the MBH in the pathogenesis of DIO. It is tempting to focus on myeloid cells in the MBH due to their position within hypothalamic CVOs, making them more amenable to drug targeting. However, currently available tools target microglia throughout the CNS. Thus, the specificity of MBH microglia in the pathogenesis of obesity compared to that of microglia in other brain regions, especially other CVOs positioned to sense circulating signals, remains intriguing.

How does innate immune activation in the MBH induce changes in neuronal activity to cause obesity? Hypothalamic neurons sense and integrate diverse signals to regulate energy homeostasis. For example, we showed that depleting microglia decreased SFA-induced neuronal injury and leptin resistance in ARC neurons and decreased SFA-induced hyperphagia^10^. However, the precise mechanism by which microglia interact with hypothalamic neurons remains largely unstudied. New tools aimed at real-time monitoring of hypothalamic neuronal function will enhance our ability to study the effects of microglia. A recent study utilizing fiber photometry and optogenetics to probe the effects of DIO on NAG neuronal circuits revealed complex changes in NAG neuronal sensitivity and downstream pathways^156^. These new tools will be central to probing how MBH innate immune activation impacts these HFD-induced changes in hypothalamic neuronal circuits.

Furthermore, it remains unclear what aspects of microglial physiology are integral to the obesity-inducing effects of the MBH innate immune response. General phagocytic function and the secretion of inflammatory cytokines have been postulated to be involved; however, recent work has shown that microglia have complex interactions with neurons, including selective phagocytosis of presynaptic structures and the induction of postsynaptic spine head filopodia^157^. Microglial phagocytosis in the context of synaptic pruning is tightly regulated. For example, the classical complement protein C1q is produced by microglia and tags synapses for elimination by microglia, both in the context of normal development and degenerative disease^158^. Another recent study showed that in the context of neuronal injury, microglia form specialized somatic junctions with neuronal cell bodies and that these connections are important for the neuroprotective role of microglia in brain injury^159^. How are these functions of microglia altered by a HFD to affect hypothalamic neurons? In addition, the heterogeneity of CNS myeloid cells has become apparent with the recent identification of disease-associated microglia (DAM) and lipid droplet-accumulating microglia (LDAM), which may play major roles in the pathogenesis of neurodegenerative diseases^160,161^. Further understanding of the heterogeneity of myeloid cells in the MBH will enhance our understanding of how microglial activation impacts hypothalamic neuronal pathways in the regulation of appetite and systemic metabolism.

With extensive ongoing work to target obesity-associated hormones, systemic inflammation, and the microbiome in the treatment of obesity, it is essential that we understand how these systemic and circulating factors influence the hypothalamic innate immune response. An improved understanding of the mechanisms by which myeloid cells in the MBH are activated by a HFD and modulate neuronal function to regulate obesity will allow more targeted experiments to understand the role of systemic factors in modulating this response.
